# Exploring health care professionals' and women's perspectives on the barriers to maternal health services: a qualitative study in Eku Town of Delta State, Nigeria

**DOI:** 10.3934/publichealth.2021012

**Published:** 2021-02-04

**Authors:** Peter Oyovwe, Gillian Woolhead

**Affiliations:** Department of Health Sciences, University of Liverpool, UK

**Keywords:** health workers, women, perspectives, barriers, maternal health services, qualitative

## Abstract

Uptake of maternal health services (MHS) have been shown to improve maternal health outcomes. In Nigeria, the maternal outcomes are classified among the poorest worldwide, despite the provision of free MHS to all pregnant women by many Nigerian state governments. The work was aimed at exploring the barriers to the uptake of MHS, from the perspectives of both women and health care professionals (HCP) in Eku Town of Delta State Nigeria, in order to gain a better understanding on views of MHS, and to guide future interventions, effective programs and policy strategies. Using an interpretivist and social constructivist epistemology approach, a qualitative study using 13 in-depth interviews were conducted. Seven women and six HCPs were recruited using purposive and snowballing sampling methods. Thematic content analysis was applied to the data. Three themes emerged. (1) Perspectives to free MHS. The majority of women preferred non-skilled Traditional Birth Attendants (TBAs) over the hospital heath service, as TBAs were viewed as more caring; with orthodox practice not being socially, culturally and religiously friendly. (2) Barriers to utilization: HCPs saw the barriers to hospital MHS as misconceptions by the women; the women mentioned the negative attitude of the HCPs and deficits in the free MHS scheme. (3) Enhancing utilization: HCPs stated that improved uptake of MHS required synergy between the community, government/hospital management through awareness, cultural/religious sensitivity, and HCP/TBA training. For the women, a change of HCPs hostile attitude and provision of more conducive hospital environment was required. There are a number of supply and demand factors that influence HCPs and women's perspectives of the uptake of MHS. Interventions and policies need to address both factors with the aim of improving the access and uptake of MHS in Nigeria.

## 1. Introduction

Maternal health outcomes (MHO) refer to the outcome of the health of women during pregnancy, childbirth and the postpartum period [1]. The major direct obstetric causes which influence MHO include ante partum and postpartum hemorrhage, pre-eclampsia/eclampsia (complications from hypertension in pregnancy), uterine rupture and obstructed labour [2]. A maternal death is said to have occurred when “a woman dies while pregnant or within 42 days after delivery or termination of pregnancy, from any cause related to or aggravated by the pregnancy or its management, but not from causes described as accidental or incidental” [2]. Maternal health outcomes, including maternal mortality ratio (MMR), are important because they are closely related to maternal health access and the quality of maternal health services (MHS) available [2].

The adoption of the Millennium Development Goals (MDG) in the year 2000, indicated that one area of policy believed to be invaluable in achieving a decrease of maternal mortality was to increase the percentage of women accessing/utilizing skilled MHS [3]. While nearly 100% of births in developed countries occur under the supervision of skilled health staff, the situation is different in sub-Saharan Africa where over 50% of births occur without the assistance of skilled health staff [4]. Sub-Saharan African women mainly depend on ‘Traditional Birth Attendants’ (TBAs), who have modest or no conventional health care training [4]. It is therefore not surprising that while the WHO recorded an estimated 830 daily deaths due to complications of pregnancy and child birth, such as hemorrhage, hypertension, infections etc.; developing countries account for 99% of these, with sub-Saharan Africa answerable for 550, representing 66% of these figures [4],[5].

In Nigeria, the maternal outcomes are among the worst in the world, with a daily loss of approximately 145 women of child bearing age and a MMR of 545 deaths per 100,000 [6], while 18 other women of these 100,000 are said to suffer various morbidities, some of which are characterized by various long term physical, psychological and socioeconomic consequences [6]. The country accounts for more than one tenth of maternal deaths worldwide [6]. This scenario represents some of the worst maternal health statistics in the world including the country being rated as the second largest contributor to the global maternal health burden [7]. Many efforts are being made by the Federal and state governments to improve maternal health outcomes, and it was in the light of these efforts that the Delta state government launched the free maternal health scheme in 2007 [8], whereby free healthcare services are provided to pregnant women [7]. It was based on the notion that financial barriers alone are responsible for the low and inequitable access to skilled MHS [9]. This is notwithstanding [10] that listed affordability, distance to facility, gender, employment status, marital status, level of income as other factors responsible for low MHS uptake [10].

Although there seems to be implications that some reductions and improvements may have taken place since the introduction of free MHS in 2007 (maternal deaths have decreased by 32 percent between 2003 and 2008) [7], women in Nigeria still face some of the highest MMR globally [7], and proportion of births attended by a skilled professional is still low [11]. This presents a major public health issue. With the identification of barriers to all health services coming from both the ‘demand’ and ‘supply’ side [12], this study was aimed at exploring the experiences of women and health care professionals (HCP) about MHS utilization, as well as the challenges in service provision.

## 2. Materials and methods

The study was conducted in Eku town of Delta State, Nigeria and the women and HCWs interviewed were selected from only Hospitals/PHCs where the Government's free MHS was ongoing. This qualitative research used semi structured interviews to help gain a rich insight into the barriers to MHS, from a woman's and a HCPs perspective. This study design provided an opportunity for knowledge sharing, as well as revealing how lay perspectives are developed over time [13].

A purposive sampling approach which required the participants to have specialist/acquired knowledge of the research issue was adopted to allow for contribution of appropriate and sufficient data in terms of relevance and depth [14]. In all cases (HCPs and Women), Snowball sampling technique where the participants with whom contact has already been made, helps the researcher in identifying other potential participants that meets the recruitment criteria was also used to recruit participants (Family Health International (FHI), 2012 Only women of 18 years and above who were pregnant or who had delivered at a non-conventional health centre were included For HCPs, the minimum qualification of nurse/midwife or medical doctor who must also be over 18 years was required and they had to have worked at the centre for at least a year.

The demographic information of the participants is presented in Table 1 and 2.

**Table 1.** Overview of reviewed articles.

Data Base	Keywords	Retrieved	Abstract reviewed	Retained	Reasons why discarded	Reasons for retaining
Discover (2014)	Barriers, health services	132	9	2	Most of the studies retrieved were on barriers to other health services.	Some of these studies are qualitative, focuses on barriers to health care services in general both at the international and continental levels.
Discover (2013–2014)	Barriers, health services	364	8	2	Most of the studies retrieved were on barriers to other health services.	Talks about barriers to maternal health services in Africa. Studies are both qualitative and quantitative
Discover (2012–2014)	Barriers, maternal health services Africa	461	10	5	Some were restricted to effect of health insurance on utilization, others are focused on only HIV/AIDS uptake.	2 qualitative, 3 quantitative, all were studies on barriers to maternal health services/facilities and all were studies done in Africa with 2 done in Nigeria.
Discover (2011–2013)	Barriers, maternal health services uptake Africa	61	9	3	Some studies restricted to uptake of PMTCT, Antiretroviral therapy, Barriers, maternal health services uptake Africa. Some already replicated elsewhere or in the proposal	2 Qualitative and 1 quantitative study, all focusing on barriers to maternal health services/facilities utilization. some studies done in rural, others in urban settings.
Google scholar (2001–2008)	Barriers, maternal health services uptake Africa	500	6	3	Some focus on barriers to surgical care, others restricted to PMTCT and financial resources. Some already replicated elsewhere or in the proposal	Talks about user perspective, contextual influences, and factors affecting utilization of maternal health services. 1 qualitative, 1 mixed and one quantitative study.
Discover	Maternal health services types	2,206	7	1	Most studies talking about maternal mortality.	Mixed study, talks about how culture affects maternal health services uptake.

**Table 2.** Barriers to accessing health services [12].

	Supply side barriers	Demand side barriers
1.	Geographic accessibility	
	Service location	Indirect household costs (transport fare)/available means of transportation
2.	Availability	
	Unqualified health staff/inadequate manpower	Awareness about the available maternal health care services
	Waiting times	Education
	Staff motivation	
	Drugs, blood and other consumables	
	Absence of opportunity/exclusion from services	
3.	Affordability	
	Cost and prices of the services on the government/provider which may include public private partnerships	Household resources/socioeconomic gradient and individual willingness to pay
		Opportunity costs
4.	Acceptability	
	Complexity of the billing system and the readiness of the user/patient to accept the bills	User/household/friends and relative expectations
	Poor staff-user relationships	Community and cultural preferences
		Poor health awareness

### 2.1. Data collection

Ethical approval was obtained both locally and from the author's institute. Data were obtained through 13 semi-structured moderate interviews involving seven women and six HCPs. These face to face interviews were conducted in English or pidgin English. These interviews sought the views of the women and HCPs which collectively resulted in a ‘thick description’ [15] of the barriers to MHS uptake. Open ended questions were used to explore the participants perspective of the barriers, coverage and uptake of MHS, as well as how these services can be improved [16].

**Table 3.** Demographic information of women participants.

Interview Number	Age	Occupation	Religion	Marital status	Education	Number of previous deliveries
WM 001	30 years	Trader	Christianity (Pentecostal)	Married	Tertiary (basic teachers certificate)	One
WM 002	21 years	Tailor	Christianity (Pentecostal)	Married	Primary school	Five
WM 003	30 years	Hairdresser (stylist)	Christianity (Pentecostal)	Married	Primary school	Four
WM 004	20 years	Trader (fruit seller)	Christianity (Pentecostal)	Married	Secondary school drop out	two
WM 005	35 years	Housewife	Christianity	Married	Secondary school drop out	Four
WM 006	27 years	Trader (food vendor)	Christianity (Pentecostal)	Married	Primary school	Seven
WM 007	32 years	Tailor	Christianity (Pentecostal)	Married	Secondary school	Five

**Table 4.** Demographic information of HCPS participants.

Interview number	Centre/hospital	Occupation	Work experience in the setting	Level of education attained	Religion	Gender	Marital status
HW 001	Hospital 1	Medical Doctor	2 years plus	Tertiary	Christian	F	Married
HW 002	Hospital 1	Medical Doctor	3 years	Tertiary	Christian	M	Married
HW 003	Hospital 1	Medical Doctor	2 years	Tertiary	Christian	M	Married
HW 004	Hospital 1	Medical Doctor	6 years	Tertiary	Christian	M	Married
HW 005	Hospital 1	Nurse/Midwife	3 years	Tertiary	Christian	F	Single
HW 006	Hospital 2	Nurse	3 years	Tertiary	Christian	F	Married

**Table 5.** Specific information about the interviews with the women and HCPs.

Interview Number	Location	Interruptions	Mode of recruitment	Duration	Translation
WM 001	TBA's	Three (interview break to allow participant have a drink, 2 interruptions by passersby/friends)	Purposively at the TBA centre	35 minutes	Pidgin English to conventional English
WM 002	Close to participant's residence	None	Purposively at the TBA centre	31 minutes	Pidgin English to conventional English
WM 003	In front of participant's residence	None	Purposively at the TBA centre	34 minutes	Pidgin English to conventional English
WM 004	In front of participant's residence	None	Snowball technique	32 minutes	Pidgin English to conventional English
WM 005	In front of participant's residence	None	Snowball technique	38 minutes	Pidgin English to conventional English
WM 006	In front of participant's residence	None	Purposively at the TBA centre	35 minutes	Pidgin English to conventional English
WM 007	Close to participant's residence	Two (first interruption by participant's kids while the other was by a phone call from her husband)	Snowball technique	38 minutes	Pidgin English to conventional English
HW 001	Participants consulting room	None	Purposive	40 minutes	None
HW 002	Verandah infront of participant's office	None	Snow ball technique	48 minutes	None
HW 003	Participants consulting room	None	Purposive	38 minutes	None
HW 004	Participants consulting room	Two (to enable researcher ensure that the recorder was working)	Purposive	40 minutes	None
HW 005	Empty Antenatal clinic hall	None	Purposive	43 minutes	None
HW 006	Participants office	None	Purposive	33 minutes	None

**Table 6.** Theme code book.

Themes	Subthemes	Codes
Perspectives of maternal health services	HCP's perspectives of free maternal health services	Aids access to free MHS. High demand Definition of “free” in MHS Hospital better than TBA (TBA-shortage of facilities, shortage of staff. Hospital recognizes complications early. TBA takes deliveries on bare floors, TBA exposes women to tetanus)
	Women's perspectives free MHS	Definition of “free in MHS” Pressure to buy items at hospital TBA better than hospital (Safety seen as being in God's hands, Hospital may be safer but TBA cares)
Barriers to utilization	HCP's perspectives on barriers to services	Lack of importance on routine checks Negative attitude of hospital staff Registration done to avoid rejection on eventual complications. Fear of CS Only basic understanding TBAs viewed as retired HCPS, Duration of bed stay Proximity of TBAs Influence of family/friends Religion/culture
	Women's views on barriers to free maternal health services	Massage not available in hospital No time for vaginal delivery in hospital Poor hospital conditions Attitude of HCPs Better care by TBA Poor hospital environment
Enhancing utilisation	Health worker's advice	Raise awareness of what is free in MHS Train TBAs on basics Government should increase manpower Raise awareness in family/friends Improve hospital and community relationships HCPs to visit TBAs
	Women's advice	Health workers should change attitude Improve hospital conditions

The interviews for the HCPs were conducted either in their private offices, or in a secluded place within the hospital/health centre premises, and for the women, the interviews were conducted in an open but secluded place around participant's home to allow for free discussion [17]. Before the participant was recruited, they were first asked a few questions to determine his/her eligibility for the interview, after which he/she was given a copy of the Participant Information Sheet (PIS), with consent duly taken before arranging an interview [18]; for the women, the PIS was often explained to them as almost all of them could not read and write. The duration of the interviews was between 28–48 minutes. All the interviews were audio taped to record the interviews. The interviews were then transcribed verbatim by the researcher on to an MS word document with the aid of transcribing software. Table 5 shows the specificities of the interviews.

The analysis of data collected was performed manually by the researcher in Word documents [19]. The transcripts were all coded systematically to classify the data. This began by formatting the data collected into code tables; colour codes were then given to each category of data [20]. Each code was then transferred into a separate word document with the relevant quotations. Similarities and differences between the quotations were identified and developed into subthemes and themes. The code book is documented in Table 6.

## 3. Results

Three overarching themes emerged from the interview data:

1. Perspectives of MHS.

2. Barriers to utilization

3. Enhancing utilization

### 3.1. Perspectives of MHS

All of the six HCPs agreed that the free MHS has helped increase patronage by the less privileged.

HW2: ‘the free maternal is fine, it helps the indigenes a lot because most of them are mainly subsistence farmers, so they cannot afford private hospitals’.

Indeed, this increase of patronage resulted in shortage of staff. Services identified by the HCPs as free in the MHS program included theatre fees, bed fees, antenatal care (ANC) registration fees, emergency fees for emergencies, like post partum hemorrhage and puerperal sepsis, the first scan, and antibiotics for the first 48 hours post surgery. However, there was a consensus that the services were not completely free. All HCPs viewed personal items, like baby wears, should be provided by the mother: *HW 1 ‘In cases of successful delivery, the necessary items include baby cloths, maternal wrappers, your flask for baby feeding. The government does not take care of these items. The only thing the government takes care of are pampers, cord clamps, pads, oxytocics, ergots hand gloves and all the drugs required. But the personal items needed by the mother and the baby are not covered/provided by the scheme’.*

All the HCPs interviewed agreed that delivery in a hospital was better than delivering at a TBA. This they based on certain parameters, including the lack of facilities and staff at the TBAs, the risk of complications not managed by a TBA, and their non observance of aseptic procedures (taking deliveries on bare floors which exposes both mother and child to infections like tetanus):

HW 5‘YES!, I think it affects it because those that patronize TBA have the disadvantage of having complications that the TBA might not be able to detect/handle; some ought to benefit from CS when they have complications like placenta previa, medical conditions like eclampsia but they end up coming here (hospital 1) at the last hour after poor management over there (at the TBA). All these seriously affect the outcome’.

The women interviewed in this study also made comments suggesting that all drugs were not free in the free MHS program, and were asked to seek their own medication, even at night. In contrast to the HCPs, they thought that all personal items should be included in the free services. They felt that if it was advertised to be free, then everything *should* be free.

WM5: ‘people are also unhappy that while the adverts/messages around suggests that the services are free, the reality suggests otherwise’.

As a consequence of some items not being provided by the free MHS, they often felt pressurized into buying items from the hospital. If they did not buy any of the additional items, most of them received insults from the staff. The additional items provided to complement the free health program were viewed as too numerous, making it more expensive than the TBA fee; this is different from the position of the HCPs who thought that the expenses are minimal.

WM 4: ‘another issue is that they keep telling you to buy one thing or the other and when you can't afford those things, they will start by complaining, then later move on to insults. This is despite the fact that they won't take good care of you’.

When asked about their preference or choice between free MHS at the hospital or the TBA, some of the women stated clearly that they preferred the TBA, the others were happy with either service. Those who made the TBA their preferred choice gave reasons like the unwelcoming attitude of the HCPs. Some held this view because of their previous experiences with HCPs or because of stories of mistreatment and abandonment in the hospital:

*WM 3 ‘that doesn't matter’. ‘I am saying so because although we hear campaigns and stories that delivery at the hospital is safer, what is the difference when someone is in labour and yet abandoned. We have heard of cases where the head of babies come out while the nurses are not on hand to take the delivery; we hear babies aspirate fluids and die at the hospital. At the TBA, they are always on hand to receive the baby and ensure everything is well’*.

Others preferred the more constant caring and understanding approach of the TBAs:

*WM 1:‘I just love this place because this mama (TBA) use to take care of us very well; rubbing (massage/physiotherapy), taking care of us by asking us questions, if I have a problem, I will happily relate it to her and because she acts promptly, I notice improvements afterwards’. ‘however at Hospital 1, it is just about collecting drugs; after seeing the Doctor’. ‘in contrast, the TBAs, will sit beside you, once you draw their attention to what is happening, they will examine you thoroughly. So I guess the nurses at the hospital are tired of doing their jobs’*.

None of the interviewees made the hospital their preferred choice. These women participants also felt the point of delivery was of no significance—what matters was one's belief in God:

*WM 2‘everything is in the hands of God. If you put your trust in God and you believe that nothing untoward will happen; then it will be well’*.

### 3.2. Barriers to utilization

According to some of the HCPs interviewed, most women who did not patronize MHS did so because they were unaware of the importance of checking vital signs:

HW 4‘So it is a question of ignorance, they don't know the benefits therein when they attend a hospital’.

Some HCPs acknowledged that the negative response by their colleagues to the women seeking MHS could be a barrier to utilization. Some are also of the opinion that the women who attended MHS, did so to ensure that there are no complications, and once that has been established, they patronized the TBA, only to return when all hope of delivering at the TBA has been disallowed:

*HW 1 ‘...then some of them are holding on to the usual patient and health worker relationship complaints that Health Workers are not receptive, i.e. t we are not welcoming them; hence, some of them still go there’. ‘People with previous scar (a previous caesarian section) will register with you, in case they are bleeding and if paradventure they are not bleeding, they deliver at the traditional birth attendant’*.

A few other HCPS admitted that a Caesarian Section (CS) can be a very traumatic experience and the thought of a high probability of going through the experience could be a reason why women crave for alternatives to hospital MHS.

*HW 2: Operation is generally a traumatic experience and many people don't like going through it and the thought of previous experience can demotivate them also, after the CS, you continue to buy drugs, the site (of operation) will still be paining you, so those are the things that motivate them (to go to the TBA)’*.

Some of the HCPs believed that convincing women to use the MHS was a phenomenal task, as the women believed that the TBAs were retired HCPs:

From the HCPs view, the TBAs are preferred because of their proximity to the patients' homes; they identified issues encountered by women who need to cross rivers before they can gain access to the hospital. For such women in their opinion, after considering transport and other logistics, the TBA might actually be cheaper. Other issues raised included the trend of women laboring for very long hours at home and presenting when the baby is almost delivered, and so would need to visit the nearest place to give birth (normally the TBA):

*HW 2: ‘Then in this Eku hinterlands, the TBA might be the only hospital (centre not hospital) that is nearby, so people that are near (around the TBA) will be taken there. Then you know most of them (the Pregnant women) wait until the fetus is almost out; by the time they can (will be able to get to the hospital), the baby would have been out; so the nearest TBA is where they are rushed to’*.

For the HCPs, friends and relatives were said to make process of utilization more cumbersome: they made patients fear surgery by misinforming them as well as taking wrong decisions especially were finance is involved. Religion and culture were also significant issues. The HCPs noted scenarios where women believed an indication for CS can be changed by faith in God or through the power of spiritual leaders. So one reason for preferring the TBA was because while the HCPs care for only physical issues, the TBAs have spiritual eyes and can therefore care for both. The culture of the Urhobos who occupy Eku community seem to regard obstetric complications as being the result of past evil deeds. Furthermore, the cultural belief among the Urhobo's is that a difficult labour or delivery requiring interventions, like CS, is a consequence of a woman's past evil deeds that requires confession to the husband and thus has a major influence on uptake.

HW 5 ‘the culture seems to suggest that any woman that undergoes a CS must have cheated on her husband or she generally has a problem with her husband and will therefore be unable to deliver normally (vaginal); hence, they will prefer to try normal vaginal delivery over there (TBA's). so the trend is such that once a woman's labour is getting prolonged) you begin to hear statements like; have you done anything (evil) to your husband? or do you have problems with him? So if there is anything (hidden), you had better confess now. So that's the little I know about it’.

Some of the women were also of the opinion that the HCPs did not allow enough time for vaginal delivery; this became worse by the way the option of surgery was suggested to them as the manner of presentation and seemed to instill fear in the patients:

*WM 3: ‘So, two months after delivery, I went to hospital 1 with my baby in my hand and showed myself to the doctor and reminded him of his earlier statement that I must deliver by CS and how God has miraculously used a TBA to deliver me through the vagina. I told him to his face that if I had come to the hospital with that labour; they (the doctors and nurses) will not even give me a chance before preparing me for surgery’*.

Other issues raised include; that the hospital environment had offensive odour, lacked water, did not make provision for meals, and had open wards such that one could see things like injured persons.

*WM 2: ‘the odour of the place is nauseating. I don't also like the odour/smell of the tablets they give’. ‘you know at the hospital you see injured people, blood on the floor and all sought of things’*.

Most of the women were unhappy with the attitude of the HCPs and some stated they were made to feel that their pregnancy was an event for regret (due to the number of children they already had).

*WM 3: ‘I believe when one goes there, just like at Hospital 1, you wouldn't like the way they may talk to you. Sometimes when you go to the hospital for antenatal, the first question may be; how many children do you have? And when you tell them say 8, it will be a recipe for insulting you. But they should consider that no one is God and after all we are better than those who abort (terminate) pregnancies. I have never aborted one myself, even though I don't take pills. But the hospital (nurses/doctors) does not understand. They (nurses/doctors) will start asking whether you want to put them (the 8 children) out for sale. ARE THEY GOD? I have decided to be patronizing the TBA’*.

Although the women saw nothing wrong with their culture and religion as regards their uptake of MHS; they however listed related issues, including their love for the TBA fee as merely a form of gratitude, non-approval of prayers at the hospital, as well as buying of baby materials before delivery as a cause of negative outcome:

*WM 3: ‘For example, in the urhobo/eku culture, when a woman is in labour, there are certain things (concoctions/oils) that she is expected to take (drink); the hospital 1 will not allow you to do such. They will instruct that no one gives you anything, touch you or even pray. ‘For example if one says go and call my pastor to pray for me or call him to bless water for me to drink; they will not allow’*.

### 3.3. Enhancing utilization

The HCPS suggestions for improvement extends government, community and hospital roles and management. They suggested that the jingle on the radio or community places about the MHS should be changed with one that properly explains what it entails. The TBAs should also be trained on the basics of obstetrics and the need for aseptic procedure, following which they can be inculcated into the free MHS. Another way of bringing in the TBAs is to first train them on who and when to refer—they can then be given incentives for bringing women to the health facility. There were also suggestions on the need for the HCPs to occasionally visit the TBAs to allow for more harmonious relationship:

*HW 3: ‘Then the TBAs too should be educated so that they can recognize when a delivery is likely to result in complications so that they can refer early to our (conventional) centres. So the TBAs, the pregnant women and the traditional rulers all need education. We need the services of the TBA but they should be trained on when to refer. For example when a woman has had a previous CS, they should not make attempt at taking the delivery because you may not be too sure of the end result. Also cases of bleeding in pregnancy even when such bleeding has stopped, they should be made to understand that there could be problems later during the delivery. Seminars can also be organized for them (TBAs)’*.

There were suggestions by the HCPs to enlighten womens' husbands on the need for skilled MHS while the women should also be educated about prolonged labour and its consequences:

HW 4: *‘and the women too should also be sensible you know you are in full labor and you have spent 10 hours in a place, why can't you move to the next level? Why won't you know there is danger? So it is part of government advocacy to do mobilization, they should also tell them that labour is not suppose to last for too long; at least we are aware that a labour that has lasted for up to 12 hours has become prolonged labour’*.

A more robust community hospital relationship should be established, with the community indigenes are advised against hostilities which could discourage HCPs from working in the community. The government perhaps had the biggest role to play; they needed to increase the available manpower as all HCPs interviewed admitted to being overwhelmed by the volume of work; they also needed to address problems of transportation and insecurity, repair roads, provide ambulance for the primary health centres, extend the free MHS to these centers and relocate those sited in remote locations.

*HW 3: ‘Then they need to address transportation and insecurity too because if a woman goes into labour at night, even if they have the transport, they may still be afraid to enter the road at that time (night) and may decide to patronize the nearest centre to have their delivery’*.

The advice from women included the need to make HCPs change their attitude towards pregnant women: they should be trained to care like the TBAs, learn to accept them just as they are and stop making pregnant women objects of ridicule:

*WM 3: ‘well, I will also tell him to help us appeal to the nurses to stop making us laughing stock and object of insults, just because we have say up to 12 children. they start asking if we are going to put them (the children) out for sale. When such a thing happens, the next time the woman gets pregnant, she becomes afraid of going there especially when she thinks of the things the hospital staff are likely going to say’*.

There is no doubt that the free maternal health program has helped uptake of MHS; its degree of coverage however needs to be clearly spelt out and further expanded. This is notwithstanding the discordance in perspectives of preferred place of delivery between the HCPs and the women. The barriers (from the perspectives of both HCPs and women) can be mitigated if the suggestions made by both HCPs and women are well implemented.

## 4. Discussion

This study captures the perspectives of both HCPs as well as women on free MHS program in Eku Community. Their combined recommendation points towards a more people centred MHS system. The HCPs perceived delivery in a hospital as better than deliveries in non-skilled centers, like the TBAs', whereas the majority of women interviewed held a contrary view, they preferred the TBA. This was based on their perception of the services rendered by HCPs as not being socially, culturally and religiously friendly. This is in comparison with the TBA services, which are viewed as more caring and pleasant by the women. This difference in perception has also been described by [21] who stated that the uptake of MHS was mainly based on socio cultural factors of the health consumers and that this related with the recipients notion of the dominant belief system, which could be contrasting with orthodox practice. It is noteworthy to state that the HCPs mentioned non observance of asepsis, delivery on bare floors, administration of massage therapy, which could result in complications like Abruptio (premature separation of the placenta from the womb) at the TBA. In contrast, the women, however, viewed the free MHS as unclean. Interestingly, this study highlighted HCPs awareness of the distinction between the caring nature of the TBAs as compared to the actions of the HCPs in free MHSs. Such admissions have not been in previous literature.

Idris et al. believed that the preference for non-skilled TBAs could be due to ignorance of the services or suspicion resulting from past experiences which may have arisen, for example, the failure of the providers to give consideration to the preferences of recipients on how the services should be executed [6]. This was further elucidated in this study where HCPs saw the gaps in the free MHS as minimal, whilst the women believed the gaps were too numerous. The gaps referred to meant that the women were being asked to buy additional items not catered for in the free MHS program and therefore put too much pressure on their meager resources. The cost identified in this study is in contrast with the [6] study which identified cost as being of little or no significance. However, both this study and the [6] study agreed that improving the quality of the services, provision of and building the capacity of the available manpower, as well as mitigating the negative attitudes of the providers/HCPs required more focus.

Like the [22] and [6] studies, this study identified barriers to uptake of MHS. These included previous unpleasant experiences of labour complications, poor quality of the MHS, inadequate manpower, cultural beliefs, distance, ignorance and suspicion, and negative attitude of the service providers. For example, the cultural belief among the Urhobo's that a difficult labour or delivery requiring interventions like CS is a consequence of a woman's past evil deeds that requires confession to the husband has a major influence on uptake. The women also saw the insistence on providing requirements like baby materials/wears as well as negative attitudes like criticizing them for having large family sizes as antagonistic to uptake.

The study also added to the pool of barriers as it identified fear of interventions like CS, the absence of opportunities for alternative therapies like massage, misrepresentation of local birth attendants for nurses and midwives, the misperception of TBAs as annexes of the hospitals, prolonged hospital stay post delivery, prolonged home stay with signs of labour, absence of transport logistics for communities across water, influence and misinformation by friends/relatives, non-conducive hospital environment, incongruity between orthodox practice and community culture and financial constraints as barriers. The women particularly noted the absence of massage therapy in orthodox practice, adjustment of breech babies in traditional practice unlike CS interventions in orthodox practice as areas of incongruity which triggers their preference for the TBA. While these incongruities seem to tally with [23]–[25] and [21] studies which suggests that a woman's perception of personal hygiene and the available health services is influenced by the prevalent cultural beliefs, none of these studies mentioned the areas of incongruity mentioned above as barriers.

Other barriers identified by the study included beliefs by some religious groups that faith in God and command by spiritual leaders were capable of changing a medical diagnosis. There was a belief in the ability of Faith based attendants to exorcise evil spirits in obstetrics complications, religious culture of rejecting interventions and the non-cultural/religious sensitivity of the MHSs. None of the literature reviewed mentioned these as barriers. However, [26] identified faith healing through the power of prophets, as well as sin, punishment from God, ancestral and evil spirits as responsible for disease and illness; they were not listed as barriers to MHS. It may therefore be safe to say that this study has found previously uncovered religious and cultural views/barriers about culture, religion and child birth.

Unlike other studies reviewed in the literature, this study has the advantage of exploring possible ways of overcoming the identified barriers. The HCPs made suggestions, such as more lucid adverts on what the free MHS entails, raising awareness on available MHS, training TBAs to acquire basic knowledge of obstetrics, inculcating TBAs to become an essential part of interventions, registration of all practicing TBAs/LBAs, giving incentives to TBAs who bring women to health facilities, Training TBAs about who and when to refer, while encouraging them to practice with caution were viewed as possible ways of enhancing uptake of MHS.

Other suggestions made by the HCPs included: increasing the manpower capacity, enlightening the men/husbands on the importance of skilled MHS, the use of women leader/traditional chief/religious leaders in awareness campaigns, holding awareness campaigns in religious/cultural meeting venues, addressing insecurity and transportation including repair of road infrastructure, encouraging cordial community-hospital relationships, educating women on the consequences of prolonged labour and encouraging HCPs to visit TBAs periodically.

The women interviewees were not left out in providing solutions to the barriers as they made suggestions like the need for HCPS to change their attitude towards users of the MHS and become more caring and polite, acceptance of MHS users by the HCPs irrespective of their deficiencies/inadequacies, provision of water and enhancing hospital environmental conditions as possible solutions.

The data from this research could have been richer if all the participants could speak English. Interpreting from Pidgin English to conventional English (which was the case for the women participants) may have affected the interpretations during transcriptions/translations [16],[27]. However, utmost care was taken when translating and the transcriptions were re-read a number of times to ensure credibility. Data were also not collected from the men who are key decision makers in health matters affecting many households and it is not clear the influence this could have had on the findings. Although the HCPs ought not to be aware of the researcher's positionality as a medical doctor, a few of them became aware and sought to know exactly who the researcher was. This knowledge of the researcher's positionality may have resulted in the HCPs not giving detailed account of all issues as they may have assumed the researcher knew about them. Such biases were mitigated by telling such participants to say everything as they know them and to assume the researcher was a novice in obstetrics. This can be seen in some interviews were the HCPs used medical terminologies with the belief that the researcher being a doctor would understand; such instances were promptly corrected with the researcher asking the interviewee to explain the meaning of such terms.

Based on our data collection and analysis of a wide range of perspectives on the barriers to MHS, we recommend certain critical factors for policy revision:

1. There is need for Government/Funding Partners to clearly delineate the degree of coverage of free MHSs.

2. Policy makers should incorporate TBAs into free MHS programs to encourage prompt referrals as well as provide for transport logistics when drawing their plans.

3. The environmental conditions around the hospital should be enhanced with water provided.

Policy makers also need to include the training of HCPs on HCP-Patient relationship as well as in cultural competence, as they have been identified from the study as major barriers to uptake. There is need to further research the cultural belief among the Urhobo's that a difficult labour or delivery requiring interventions like CS is a consequence of a woman's past evil deeds which requires confession to the husband as it has a major influence on uptake.

4. We also recommend that well instituted organizational norms such as those that help reduce burn out/pressure for time at the work place could assist in reducing the treatment of patients with disdain. ‘reciprocity of likeness' from patients is also capable of influencing attitudes and behaviors of HCPs [28].

5. We limited our study to the perspectives of women and HCPs only; We hope future studies will include perspectives from TBAs, Faith based attendants (FBAs) and Local birth attendants (LBAs) whose services were referred to severally as they may have added further insights to the barriers.

## 5. Conclusion

While previous studies on barriers to uptake focused on the views of the users, this study, using qualitative methods, has made unique contributions by exploring not just the perspectives of the users but those of the providers as well. The study agrees with barriers listed in previous studies but listed new barriers as well; thus contributing to knowledge on why women could fail/refuse to deliver at a conventional facility even while such services are free.

The study uncovers assumptions by the providers/HCPs that they are doing the right thing, without taking into cognizance the views of the recipients of the services. Inevitably, such assumptions could only result in poor outcomes in terms of uptake of the services. It is clear from the study that the recipients have justifiable reasons for not patronizing the free services.

A review of all programs/interventions towards becoming more socio-culturally and religiously friendly could help improve uptake of MHS.[28] also opined that well instituted organizational norms such as those that help reduce burn out/pressure for time at the work place could assist in reducing the treatment of patients with disdain. They also identified ‘reciprocity of likeness' from patients as capable of influencing attitudes and behaviors of HCPs.

It may therefore be appropriate to consider this study as health promotion friendly, as it is targeted at enabling women take control of and improve their health through uptake of MHS.
